# Bone marrow-derived mesenchymal stem cells promote growth and angiogenesis of breast and prostate tumors

**DOI:** 10.1186/scrt221

**Published:** 2013-06-13

**Authors:** Ting Zhang, Yuk Wai Lee, Yun Feng Rui, Tin Yan Cheng, Xiao Hua Jiang, Gang Li

**Affiliations:** 1 Department of Orthopaedics and Traumatology, The Chinese University of Hong Kong, Prince of Wales Hospital, 30-32 Ngan Shing Street, Shatin, New Territories, Hong Kong, PR China; 2 Stem Cells and Regeneration Program, School of Biomedical Sciences, Li Ka Shing Institute of Health Sciences, The Chinese University of Hong Kong, Prince of Wales Hospital, 30-32 Ngan Shing Street, Shatin, New Territories, Hong Kong SAR, PR China; 3 Department of Orthopaedics, Zhongda Hospital, School of Medicine, Southeast University, Nanjing, Jiangsu, PR China; 4 The Chinese University of Hong Kong Shenzhen Research Institute, Shenzhen, PR China

**Keywords:** Mesenchymal Stem Cells, Tumor Growth, Angiogenesis

## Abstract

**Introduction:**

Mesenchymal stem cells (MSCs) are known to migrate to tumor tissues. This behavior of MSCs has been exploited as a tumor-targeting strategy for cell-based cancer therapy. However, the effects of MSCs on tumor growth are controversial. This study was designed to determine the effect of MSCs on the growth of breast and prostate tumors.

**Methods:**

Bone marrow-derived MSCs (BM-MSCs) were isolated and characterized. Effects of BM-MSCs on tumor cell proliferation were analyzed in a co-culture system with mouse breast cancer cell 4T1 or human prostate cancer cell DU145. Tumor cells were injected into nude mice subcutaneously either alone or coupled with BM-MSCs. The expression of cell proliferation and angiogenesis-related proteins in tumor tissues were immunofluorescence analyzed. The angiogenic effect of BM-MSCs was detected using a tube formation assay. The effects of the crosstalk between tumor cells and BM-MSCs on expression of angiogenesis related markers were examined by immunofluorescence and real-time PCR.

**Results:**

Both co-culturing with mice BM-MSCs (mBM-MSCs) and treatment with mBM-MSC-conditioned medium enhanced the growth of 4T1 cells. Co-injection of 4T1 cells and mBM-MSCs into nude mice led to increased tumor size compared with injection of 4T1 cells alone. Similar experiments using DU145 cells and human BM-MSCs (hBM-MSCs) instead of 4T1 cells and mBM-MSCs obtained consistent results. Compared with tumors induced by injection of tumor cells alone, the blood vessel area was greater in tumors from co-injection of tumor cells with BM-MSCs, which correlated with decreased central tumor necrosis and increased tumor cell proliferation. Furthermore, both conditioned medium from hBM-MSCs alone and co-cultures of hBM-MSCs with DU145 cells were able to promote tube formation ability of human umbilical vein endothelial cells. When hBM-MSCs are exposed to the DU145 cell environment, the expression of markers associated with neovascularization (macrophage inflammatory protein-2, vascular endothelial growth factor, transforming growth factor-beta and IL-6) was increased.

**Conclusion:**

These results indicate that BM-MSCs promote tumor growth and suggest that the crosstalk between tumor cells and BM-MSCs increased the expression of pro-angiogenic factors, which may have induced tumor cell proliferation and angiogenesis thereby increasing solid tumor growth.

## Introduction

Tumor development and progression has been recognized as the product of an evolving crosstalk between different cell types within the tumor and its surrounding supporting tissue, or tumor stroma [1-3]. The tumor stroma is composed of extracellular matrix as well as a multitude of cell types such as fibroblasts and myofibroblasts, immune and inflammatory cells, adipocytes, pericytes and endothelial cells of the blood and lymphatic circulation [2]. The mutual interactions between tumor cells and stromal cells via direct contact or through the production of growth factors, cytokines and chemokines in a paracrine manner are thought to modulate tumor expansion, invasion, metastasis and angiogenesis [2,4-9]. As one of the most crucial components of the tumor microenvironment, carcinoma-associated fibroblasts (CAFs) have been shown to precede the onset of invasion and promote tumor cell survival as well as migratory properties [10]. Various different mobilized cell types including normal fibroblasts, preadipocytes, epithelial cells and smooth muscle cells have been shown to be the sources of CAFs [11]. Accumulating evidence from both human and mouse tumor models suggests that bone marrow-derived mesenchymal stem cells (BM-MSCs) have a significant contribution to CAF and myofibroblast populations within the tumor stroma [12-15].

Mesenchymal stem cells (MSCs) are multipotent adult stem cells of mesodermal germ layer origin that possess an innate ability for self-renewal and are capable of differentiating into a variety of mesodermal lineages, including chondrocytes, osteoblasts and adipocytes under proper experimental conditions *in vitro* and *in vivo*[16,17]. The multilineage potential of MSCs plays an important role in wound healing and tissue regeneration through differentiation and the release of important growth factors and cytokines [18-20]. Secretion of chemokines/cytokines from the neoplasm or inflammatory tissues such as vascular endothelial growth factor (VEGF), transforming growth factors (TGFs), fibroblast growth factors (FGF), platelet-derived growth factors (PDGF) and interleukin-8 (IL-8) is known to promote the migration of MSCs from the bone marrow [21,22]. Tumor/cancer is considered a wound that never heals and tumor microenvironments have many similarities with the tissue repair processes that attract specific homing of MSCs [23,24]. The tumor homing properties of MSCs made them ideal candidates as anti-tumor agent delivery vehicles [25] and also attracted increased interest in understanding the role and fate of MSCs in tumor development and growth. Several studies have indicated that MSCs could enhance tumor growth and metastasis [26,27]. Mishra and colleagues have demonstrated that, by prolonged exposure to tumor cell conditioned medium, MSCs could be activated, differentiated into CAFs and become part of the tumor microenvironment [12]. To the contrary, there are studies indicating that MSCs also display intrinsic anticancer activities such as those in an *in vivo* model of Kaposi's sarcoma [28].

In most studies regarding the effect of MSCs on tumors, human tumor cells and human MSCs were used in mouse models. The stromal cells in this tumor xenograft model are thus from two different species. There may be some unknown interactions between the human and mouse cells that would affect the analysis. In this study, in addition to studying the effect of human bone marrow-derived mesenchymal stem cells (hBM-MSCs) on human prostate cancer growth, the mouse mammary tumor cell line 4T1 was selected to study the effect of mouse bone marrow-derived mesenchymal stem cells (mBM-MSCs) on tumor growth. For the latter, all cells used are of mouse origin and one can therefore interpret the results more clearly. We used luciferase-labeled tumor cells and co-cultured methods to access the tumor cell growth *in vitro*. We found that in both co-culture with mBM-MSCs and exposure to conditioned medium of mBM-MSCs, the proliferation of Luc-4T1 cells was promoted *in vitro*. Furthermore, with co-injection of mBM-MSCs with 4T1 cells into the nude mice, the tumor growth was enhanced. For the effect of hBM-MSCs on the growth of DU145 cells, we obtained consistent results. The underlying mechanism was also studied. We found that BM-MSCs could promote tumor growth through enhanced angiogenesis.

## Materials and methods

### Cell culture

The 4T1 mouse mammary tumor cell line was purchased from American Type Culture Collection and cultured in alpha-minimum essential medium (α-MEM, Manassas, Virginia, US) supplemented with 10% fetal bovine serum (FBS) and 1% penicillin–streptomycin–neomycin (complete culture medium; all from Invitrogen Corporation, Carlsbad, CA, USA). Human prostate cancer cell line DU145 was obtained from American Type Culture Collection and grown in RPMI 1640 medium supplemented with 10% FBS and 1% penicillin–streptomycin–neomycin.

Mouse skin fibroblasts were isolated from dorsal skin and cultured in complete α-MEM medium. For the primary culture of mBM-MSCs, bone marrow was flushed and harvest from the femur of 6-week-old to 8-week-old FVB mice. The cell suspension was then filtered and the bone marrow cells cultured in a 100 mm culture dish in α-MEM containing 20% FBS. After 3 days the nonadherent cells were removed by changing the medium; after an additional 4 to 7 days the culture becomes confluent, and when it reaches 70 to 80% confluence the adherent cells were trypsinized and subcultured. After three to five passages, a homogeneous cell population was obtained and used for further expansion and characterization. The mBM-MSCs were used at passage 5 in this study.

Human fetal bone marrow stem cells (hBM-MSCs) were donated from the Stem Cell Bank of the Prince of Wales Hospital of the Chinese University of Hong Kong. Human ethics approval was obtained from the Joint Chinese University of Hong Kong-New Territories East Cluster Clinical Research Ethics Committee (ethical approval code: CRE-2011.383). Informed written consent form was approved by the Clinical Research Ethics Committee and signed by donor before sample collection. 4T1 cells, DU145 cells stably expressing luciferase (Luc-4T1, Luc-DU145) and hBM-MSCs stably expressing GFP were kept in our laboratory.

### Characterization of mouse bone marrow-derived MSCs

To identify the isolated cells, cell surface markers and differential potential were analyzed. Flow cytometric analysis was applied for examining the expression of surface antigens of the cells. Briefly, cells at passage 5 were harvested and cell suspensions containing 1 × 10^5^ cells were stained with the fluorescence conjugated antibodies phycoerythrin-conjugated rat anti-mouse CD44, phycoerythrin-conjugated rat anti-mouse Sca-1, fluorescein isothiocyanate-conjugated rat anti-mouse CD45 and fluorescein isothiocyanate-conjugated rat anti-mouse CD34 (BD Pharmingen, Franklin Lakes, NJ, USA) for 1 hour at 4°C. After washing with PBS, the cells were resuspended in 0.5 ml stain buffer (BD Pharmingen) for flow cytometric analysis. Nonspecific background signals were measured by incubating the cells with the appropriate isotype control antibodies. The percentage of cells with a positive signal and the mean geometric fluorescence value of the positive population were calculated using the WinMDI Version 2.9 program (The Scripps Research Institute, La Jolla, CA, USA). For osteogenic differentiation, the cells at passage 5 were seeded in a six-well plate at a density of 4 × 10^3^ cells/cm^2^ and cultured in the base complete medium for 2 or 3 days until they reached 80% confluence. The medium was then removed and replaced by osteogenic induction medium, which was complete medium supplemented with 1 nM dexamethasone, 50 mM l-ascorbic acid-2-phosphate, and 20 mM β-glycerolphosphate for 14 days (all from Sigma-Aldrich, St Louis, MO, USA). The induction medium was changed every 3 days. Cells cultured in the base complete medium were used as a negative control. Alizarin Red S staining is used for the assessment of calcium compound formation. For adipogenic differentiation, cells were plated at a density of 4 × 10^3^ cells/cm^2^ in a six-well plate and cultured in base complete medium for 2 or 3 days until they reached 80% confluence. Afterwards the medium was removed and replaced by adipogenic medium, which was complete medium supplemented with 500 nM dexamethasone, 0.5 mM isobutyl-methylxanthine, 50 mM indomethacin, and 10 mg/ml insulin (all from Sigma-Aldrich). Cells cultured in the base complete medium served as a negative control. After 14 days of culture, 2% (wt/vol) Oil Red-O solution (Sigma-Aldrich) was applied to identify the presence of lipid-rich vacuoles. For chondrogenic differentiation, about 8 × 10^5^ cells were pelleted into a micromass by centrifugation at 450 × *g* for 10 minutes in a 15 ml conical polypropylene tube and cultured in complete basal medium or chondrogenic medium, which contained LG-DMEM supplemented with 10 ng/ml TGF-β1 (Gibco, Invitrogen Corporation), 10^–7^ M dexamethasone, 50 μg/ml ascorbate-2-phosphate, 40 μg/ml proline, 100 μg/ml pyruvate (all from Sigma-Aldrich), and 1:100 diluted BD™-ITS Universal Culture Supplement Premix (Becton Dickinson, Franklin Lakes, NJ, USA). At day 21, the pellet was fixed for safranin-O/fast green staining.

### *In vitro* cell proliferation assays

For investigation of the effect of BM-MSCs on proliferation of tumor cells, luciferase-labeled tumor cell line Luc-4T1 was co-cultured with either 4T1, mouse skin fibroblasts or mBM-MSCs in a 96-well black plate at a ratio of 1:1 in a density of 1.0 × 10^4^/well in α-MEM containing 1% FBS. Similar experiments were conducted for Luc-DU145. Tumor cell proliferation was examined every 12 hours for a 72-hour period using the IVIS 200 in Vivo Imaging System (PerkinElmer, Waltham, MA, USA) according to the manufacturer’s instructions. Briefly, after removing the medium, the fresh medium containing d-luciferin (Biosynth, Itasca, IL, USA) at a concentration of 150 μg/ml was added. Prior to imaging examination, the plate was incubated at 37°C for 10 minutes. Bioluminescent images were acquired and the bioluminescent intensity was quantified in photons/second using Living Image 2.5 software (PerkinElmer) accordingly. For analyzing the dose–response effect of BM-MSCs on tumor cell proliferation, Luc-4T1 or Luc-DU145 cells were cultured alone or incubated with BM-MSCs at ratios of 1:0.2, 1:0.5, 1:1, 1:2, 1:5, 1:10 and 1:15. At the same time, Luc-4T1 or Luc-DU145 cells were incubated alone or in combination with mouse skin fibroblasts at different ratios as a control. After 48 hours of culture, the bioluminescent images were acquired and the bioluminescent intensity was quantified.

To investigate the effect of conditioned medium from BM-MSCs on tumor cell proliferation, conditioned medium was collected from mBM-MSCs and hBM-MSCs during the logarithmic growth phase. Briefly, BM-MSCs were plated in a 75 cm^2^ flask in 12 ml complete medium for 18 to 24 hours of culture, and when they reached preconfluence the medium was changed and the cells were rinsed in 1× PBS twice and cultured in fresh medium containing 1% FBS for an additional 3 days. The medium was then centrifuged at 1,000 × *g* for 10 minutes at 4°C for clarifying and the supernatant regarded as conditioned medium was collected and stored at −80°C for future use. To assess the tumor cell proliferation, Luc-4T1 or Luc-DU145 cells were seeded at 5.0 × 10^3^ cells/well in a black 96-well plate in complete medium for 12 hours. Afterwards the medium was replaced by either conditioned medium collected from BM-MSCs or α-MEM containing 1% FBS. Tumor cell proliferation was assessed every 12 hours for a 96-hour period using the IVIS 200 in Vivo Imaging System. Tumor cell proliferation in the presence of conditioned medium from BM-MSCs was also assessed using the BrdU assay kit (Roche Applied Science, Penzberg, Upper Bavaria, Germany) according to the manufacturer’s instructions. 4T1 or DU145 cells were seeded at a density of 4 × 10^3^ cells/cm^2^ in a 96-well plate and conditioned medium or α-MEM/1% FBS was replaced after 12 hours. After labeling with BrdU for 2 hours by adding BrdU labeling reagent into the medium, the cells were then fixed and incubated with anti-BrdU antibody that is peroxidase conjugated for 1 hour. Followed by washing with washing buffer, the substrate solution TMB was added and incubated for 5 minutes. The absorbance at 450 nm was measured and reported.

### *In vivo* tumor growth analysis

All experiments were approved by the Animal Research Ethics Committee of the authors’ institution. For assessing the effect of BM-MSCs on tumor growth *in vivo*, all nude mice were randomly divided into the following groups (*n* = 6) for transplanted cells through subcutaneous injection: 4T1 cells alone (2.0 × 10^6^); 4T1 cells (2.0 × 10^6^) mixed with mBM-MSCs (2.0 × 10^6^) at a ratio of 1:1; mBM-MSCs alone (2.0 × 10^6^); 4T1 cells (2.0 × 10^6^) mixed with mouse skin fibroblasts (2.0 × 10^6^) at a ratio of 1:1; DU145 cells alone (2.0 × 10^6^); DU145 cells (2.0 × 10^6^) mixed with hBM-MSCs (2.0 × 10^6^) at a ratio of 1:1; hBM-MSCs alone; and DU145 cells (2.0 × 10^6^) mixed with mouse skin fibroblasts (2.0 × 10^6^) at a ratio of 1:1. The cells were suspended in 200 μl 1× PBS and injected subcutaneously into the dorsal sides of nude mice. Beginning 5 days after transplantation, the size of the tumor was measured with a caliper and the tumor volume was calculated using the following formula:

$$\begin{array}{ll} \;\mathrm{Tumor}\;\mathrm{volume}\left(mm^{3}\right)= & 0.52\times \mathrm{width}{\left(\mathrm{mm}\right)}^{2}\\ & \times \mathrm{length}\left(\mathrm{mm}\right) \end{array}$$

After 21 days, nude mice were sacrificed using 20% overdose pentobarbital. The tumors were removed and cut into two pieces. One-half of the tumors were embedded in Optimal Cutting Temperature medium, frozen in liquid nitrogen, and stored at −80°C. The other tumors were fixed in formalin, dehydrated through increasing concentrations of ethanol and embedded in paraffin for immunochemistry.

### Immunofluorescence staining

For immunofluorescence staining, the frozen samples were cut into 5-μm sections using a cryostat microtome (Leica, Wetzlar, Hesse, Germany). The slides were fixed in 4% paraformaldehyde in PBS (pH 7.4) for 15 minutes at room temperature and washed with cold PBS. After treatment with PBS containing 0.25% Triton X-100 for 10 minutes for permeabilization, the samples were incubated with 5% normal goat serum/1% BSA/PBS for 30 minutes to block unspecific binding of the antibodies. Subsequently the slides were incubated with primary antibodies specific to CD31 (Abcam, Cambridge, UK) at 1:50, alpha smooth muscle actin (α-SMA; Abcam) at 1:100 and Ki67 (Abcam) at 1:200 overnight at 4°C, followed by PBS washing three times. Alexa Fluor 488-conjugated secondary antibody or Alexa Fluor 594-conjugated secondary antibody was incubated for 1 hour at room temperature (Invitrogen). ProLong Gold antifade reagent with DAPI (Invitrogen) was applied to mount and counterstain the slides. All of the fluorescence pictures were captured and analyzed under a fluorescent microscope (Zeiss-spot; Carl Zeiss MicroImaging GmbH, Jena, Thuringia, Germany). For quantification of the microvessel area, 10 fields at 200× were randomly selected from sections stained with CD31 antibody and the vessel area was measured using ImageJ software [29]. For assessment of cell proliferation *in vivo*, the percentage of Ki-67-positive cells was calculated using the ImageJ software.

For cell immunofluorescence staining, hBM-MSCs were seeded in sterilized glass coverslips placed in a six-well plate. The cells were starved for 24 hours and treated with conditioned medium for 7 days or not. After removing the medium, the cells were fixed in 4% paraformaldehyde for 10 minutes at room temperature, washed with cold PBS twice, incubated with PBS containing 0.25% Triton X-100 for 10 minutes, blocked with 5% normal goat serum/1% BSA/PBS for 30 minutes and then incubated with primary antibodies for 2 hours at room temperature. Cells were immunostained for VEGF, TGF-β and IL-6 (all from Santa Cruz, Dallas, Texas, USA) at 1:100. Following PBS washing, the cells were incubated with Alexa Fluor 488-conjugated secondary antibody or Alexa Fluor 594-conjugated secondary antibody. Finally, all cells were mounted and counterstained with ProLong Gold antifade reagent with DAPI.

### Immunochemistry

For immunochemistry, the paraffin blocking was cut into 5 μm sections and then deparaffinized and redehydrated. Subsequently the sections were incubated in 3% H_2_O_2_ solution in methanol at room temperature for 10 minutes to block endogenous peroxidase activity and, after rinsing in PBS twice, antigen retrieval was performed by arranging the sides in a stain container containing 10 mM citrate buffer (pH 6.0) at 95°C for 15 minutes. After cooling and washing, blocking buffer (5% normal goat serum/1% BSA/PBS) was added and incubated for 30 minutes at room temperature. The sections were then incubated with diluted primary antibodies anti-GFP (Abcam) at 1:1,000 overnight at 4°C. Followed by washing three times, horseradish peroxidase-conjugated rabbit anti-goat secondary antibodies were applied for 1 hour at room temperature. The substrate 3,3′-diaminobenzidine (Dako, Glostrup, Denmark) was used for color development. Sections were then rinsed in running tap water and counterstained with hematoxylin. The primary antibodies replaced by antibody diluent served as a negative control. At least five sections from each sample were analyzed.

### Exposure of human bone marrow-derived MSCs to conditioned medium of tumor cells

DU145 cells were plated in 75 cm^2^ flasks in α-MEM containing 1% FBS. The tumor cell conditioned medium was harvested during their logarithmic growth phase, centrifuged at 1,000 × *g* for 10 minutes at 4°C for clarifying and stored at −80°C for subsequent use. Before exposure to conditioned medium, hBM-MSCs were serum-starved by cultured in serum-free α-MEM for 24 hours. Afterwards BM-MSCs were cultured in DU145 conditioned medium or α-MEM/1% FBS and the medium was changed every 2 days for the entire culturing period (7 days).

### Tube formation assays

For assessment of angiogenic activity of interaction between BM-MSCs and tumor cells *in vivo*, tube formation assays were performed. Matrigel (100 μl; BD Pharmingen) was paved on a well of a 96-well plate and incubated for 1 hour at 37°C to allow the gel to solidify. Then 1.5 × 10^4^ human umbilical vein endothelial cells were seeded in: base culture medium (α-MEM); hBM-MSC conditioned medium; DU145 cell conditioned medium; and hBM-MSCs co-cultured with DU145 conditioned medium at a final volume of 100 μl. All assays were independently performed six times for each group. After incubation for 8 hours, the cells were visualized using a light microscope. Endothelial tubule length was quantitatively analyzed using Image-Pro Plus software 6.0 (Media Cybernetics, Rockville, MD, USA).

### RNA extraction and real-time quantitative PCR

The expression level of angiogenic factor was analyzed by quantitative real-time PCR. hBM-MSCs were co-cultured with DU145 using noncontacting co-culture transwell systems or were treated by conditioned medium from DU145. Cells cultured in α-MEM served as a control. After 7 days culturing, cells were harvested and RNA was extracted using an RNA extraction mini kit (Life Technologies, Carlsbad, CA, USA) according to the manufacturer’s instructions. Then 800 ng total RNA of each sample was reverse transcribed to cDNA by M-MLV Reverse Transcriptase (Life Technologies). Real-time PCR assays were performed on the ABI StepOne Plus system using Power SYBR® Green PCR Master Mix (Life Technologies) with 4 ng (10-fold dilutions) standard cDNA and 400 nM specific primers for IL-6, TGF-β, VEGF and macrophage inflammatory protein-2 (MIP-2). The sequences were as follows: IL-6, 5′-GGTACATCCTCGACGGCATCT-3′ (forward) and 5′-GTGCCTCTTTGCTGCTTTCAC-3′ (reverse); TGF-β, 5′-CCCAGCATCTGCAAAGCTC-3′ (forward) and 5′-GTCAATGTACAGCTGCCGCA-3′ (reverse); VEGF, 5′-CTACCTCCACCATGCCAAGT-3′ (forward) and 5′-GCAGTAGCTGCGCTGATAGA-3′ (reverse); and MIP-2, 5′-CGCCCAAACCGAAGTCAT-3′ (forward) and 5′-GATTTGCCATTTTTCAGCATCTTT-3′ (reverse). The amplification was performed under the following conditions: one cycle of denaturation at 95°C for 10 minutes, 40 cycles of denaturation at 95°C for 30 seconds, annealing at 60°C for 30 seconds, and extension 72°C for 30 seconds. Fluorescence data were acquired at the end of each annealing step. Finally, a melting curve was generated by increasing the temperature from 65 to 95°C. The expression of target gene was measured relative to that of GAPDH as housekeeping gene. The primers for amplify GAPDH were: 5′-AGGGCTGCTTTTAACTCTGGT-3′ (forward) and 5′-CCCCACTTGATTTTGGAGGGA-3′ (reverse). All samples were performed in triplicate.

### Statistical analysis

Results are expressed as the mean ± standard deviation. Data were analyzed with GraphPad Prism statistical software 6.0 (GraphPad Software, La Jolla, CA, USA) using either one-way analysis of variance followed by Tukey’s *post-hoc* test or Student’s *t* test where appropriate. *P* <0.05 was considered statistically significant.

## Results

### Characterization of bone marrow-derived MSCs

**Figure 1:**
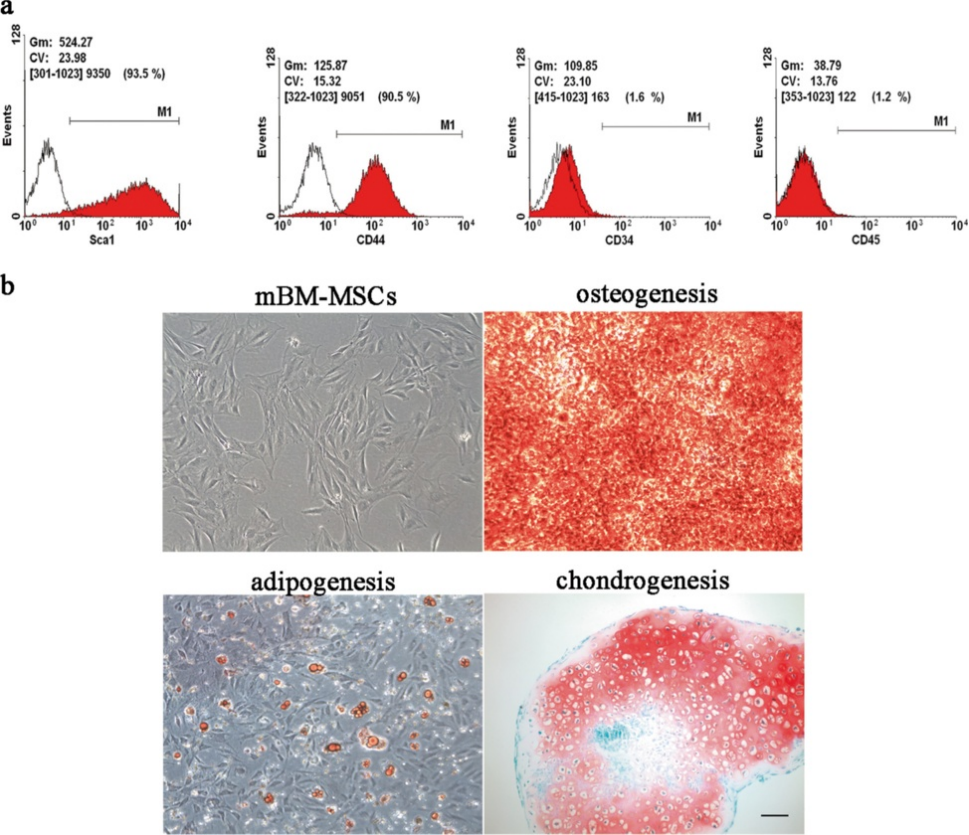
**Characterization of mouse bone marrow-derived mesenchymal stem cells.** (**a**) Flow cytometric analysis showing the expressing of cell surface markers Sca1, CD44, CD34 and CD45. (**b**) Spindle-shaped morphology of cells that appear at passage 5 (upper left); the cells differentiated into mineralizing cells stained with alizarin red (upper right); adipogenesis of cells was stained with Oil-red-O (lower left); and accumulation of sulfated glycosaminoglycan matrix deposition was visualized by Safarin-O staining at 3 weeks of chondrogenic induction (the lower right). Scale bar, 100 μm. mBM-MSCs, mouse bone marrow-derived mesenchymal stem cells.

mBM-MSCs were established from FVB mice of 6 weeks old and were subjected to flow cytometry as well as a differentiation assay *in vitro*. The mBM-MSCs exhibited fibroblast-like spindle-shaped morphology in culture at passage 5 (Figure 1b) and the surface marker expression on these cells was characterized. The flow cytometry results revealed that the BM-MSCs were positive for Sca-1 and CD44, which are characteristically expressed on MSCs, but negative for CD34 and CD45 (Figure 1a). In order to examine whether BM-MSCs show multipotent differentiation potential *in vitro*, adipogenic, osteogenic and chondrogenic induction were performed. Fourteen days after adipogenic induction, oil red O staining results showed that the BM-MSCs were committed toward adipogenic lineage and filled with lipid-rich vacuoles (Figure 1b). Following osteogenic induction, as shown by the alizarin red staining result in Figure 1b, there was mineralized extracellular matrix generated in culture, suggesting osteoblastic differentiation (Figure 1b). Safranin-O/fast green staining for sulfated glycosaminoglycan matrix deposition of pellets showed chondrogenic differentiation of BM-MSCs in a 21-day pellet culture with induction medium (Figure 1b). Combined, these data indicated that BM-MSCs are indeed MSCs from bone marrow.

### Co-culture with bone marrow-derived MSCs promotes proliferation of tumor cells *in vitro*

**Figure 2:**
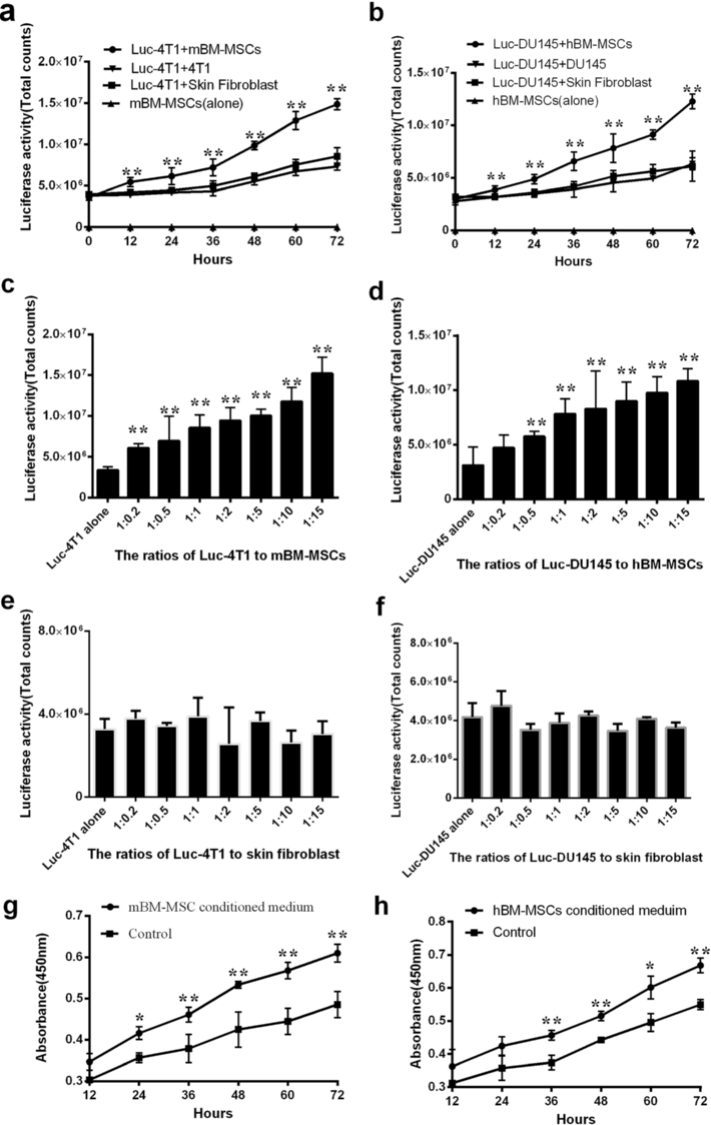
**Bone marrow-derived mesenchymal stem cells enhance breast and prostate cancer cell proliferation *in vitro.*** (**a**) Luc-4T1 cells were co-cultured with mouse bone marrow-derived mesenchymal stem cells (mBM-MSCs), 4T1 cells or mouse skin fibroblasts and (**b**) Luc-DU145 cells were cultured with human bone marrow-derived mesenchymal stem cells (hBM-MSCs), DU145 cells or mouse skin fibroblasts at the ratio of 1:1. Luciferase activities were measured every 12 hours for a 72-hour period to determine growth of tumor cells in the co-culture assay (*n* = 6 per group; **P <0.01). (**c**) Luc-4T1 cells were incubated with mBM-MSCs and (**d**) Luc-DU145 cells were incubated with hBM-MSCs at different ratios. (**e**) Luc-4T1 cells were incubated with mouse skin fibroblasts and (**f**) Luc-DU145 cells were incubated with mouse skin fibroblasts at different ratios. Luciferase activities were measured after 48 hours (*n* = 6 per group; **P <0.01). (**g**) 4T1 cells were cultured in mBM-MSC conditioned medium and (**h**) DU145 cells were cultured in hBM-MSC conditioned medium for 72 hours. BrdU assay was performed to measure the proliferation of tumor cells at the indicated time points (*n* = 6 per group; *P <0.05, **P <0.01).

To investigate whether BM-MSCs could promote proliferation of the tumor cells *in vitro*, the growth of Luc-4T1 cells co-cultured with mBM-MSCs were compared with those of Luc-4T1 mixed with mouse skin fibroblasts or 4T1 cells. For DU145, similar experiments were conducted. These cells were incubated in black 96-well plates and luciferase activities were measured at different time points. There are good correlations between cell numbers and bioluminescence in the cells transduced with the luciferase gene using the In Vivo imaging system IVIS 200 as we reported earlier [30]. Significantly, co-culture with mBM-MSCs, but not with 4T1 or fibroblasts, enhanced the proliferation of Luc-4T1 after 72 hours (*P* <0.01) (Figure 2a); consistently, we found that the growth of Luc-DU145 cells was also enhanced when co-cultured with hBM-MSCs, but not with DU145 cells or mouse skin fibroblasts (*P* <0.01) (Figure 2b). These results showed that when co-cultured with BM-MSCs, the proliferation of Luc-4T1 or Luc-DU145 could be promoted *in vitro*. To determine whether the dosage of MSCs could affect the proliferation of tumor cells, Luc-4T1 with mBM-MSCs or Luc-DU145 with hBM-MSCs were co-cultured at different ratios for 48 hours. The results indicated that Luc-4T1 cells and mBM-MSCs at 1:1 and 1:15 ratios exhibited 2.52-fold (*P* <0.01) and 4.48-fold (*P* <0.01) greater Luc-4T1 cell number after 48 hours (Figure 2c). The results also showed that there was 2.50-fold (*P* <0.01) and 3.46-fold (*P* <0.01) increase in proliferation of Luc-DU145 when the ratio of Luc-DU145 and hBM-MSCs were 1:1 and 1:15 res-pectively (Figure 2d). However, when tumor cells incubated with fibroblasts at different ratios, no significant promoting effect was observed during 48 hours of incubation (Figure 2e,f). On the basis of these data, we found that the proliferation of Luc-4T1 and Luc-DU145 cell increases were in accordance with the number of BM-MSCs that presented in the co-culture system.

To further evaluate the effect of conditioned medium from BM-MSCs on the growth of tumor cells, conditioned medium was obtained from mBM-MSCs or hBM-MSCs and its effect on 4T1 or DU145 cell proliferation was tested respectively. Results from the BrdU assay indicated that, compared with culture in α-MEM, there was a 1.22-fold (*P* <0.01) increase in proliferation of 4T1 cells (Figure 2g) and a 1.17-fold increase (*P* <0.01) in DU145 cells (Figure 2h) in the presence of conditioned medium from mBM-MSCs or hBM-MSCs.

### Bone marrow-derived MSCs promote tumor growth *in vivo*

**Figure 3:**
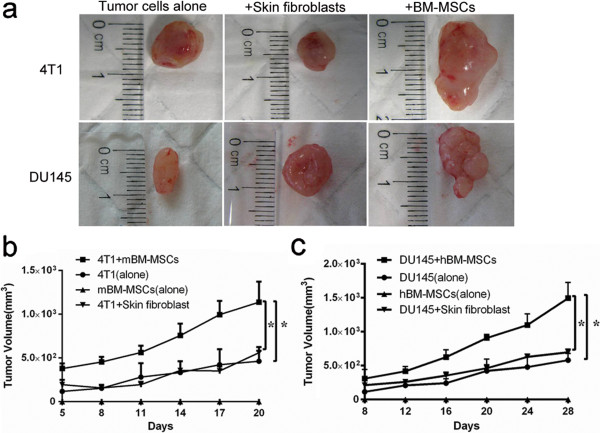
**Bone marrow-derived mesenchymal stem cells promote tumor growth in nude mice.** (**a**) Representative photographs of 4T1 and DU145 tumors generated from nude mice injected with tumor cells alone, co-injected with mouse skin fibroblasts, or bone marrow-derived mesenchymal stem cells (BM-MSCs). (**b**) Mouse bone marrow-derived mesenchymal stem cells (mBM-MSCs) alone and 4T1 cells along with or without mBM-MSCs/skin fibroblasts were injected and the tumor size was measured and calculated every 3 days *(n* = 6 per group; *P <0.05). (**c**) Human bone marrow-derived mesenchymal stem cells (hBM-MSCs) alone and DU145 cells along with or without hBM-MSCs/skin fibroblasts were injected and tumor size was measured and calculated every 4 days (*n* = 6 per group; *P <0.05).

To investigate *in vivo* effects of BM-MSCs on tumor growth, mBM-MSCs and 4T1 cells or hBM-MSCs and DU145 cells were mixed together and implanted into nude mice respectively. At the same time, tumor cells were also co-injected with mouse skin fibroblasts with an equal number of tumor cells plus BM-MSCs. At day 20 post tumor inoculation, the tumors generated in nude mice injected with mBM-MSCs and 4T1 cells exhibited a 2.45-fold increase in tumor volume compared with tumors from injected 4T1 cells alone (*P* <0.05) (Figure 3a,b). At day 28 post tumor inoculations, mice injected with hBM-MSCs and DU145 showed 2.56-fold (*P* <0.05) greater tumor volume than mice injected with DU145 alone (Figure 3a,c). Moreover, the BM-MSC-induced increase in the tumor growth was significantly more than that when mixed with fibroblasts (*P* <0.05) and there was no statistical difference between the fibroblast group and the tumor cells alone group (Figure 3). Mice injected with mBM-MSCs or hBM-MSCs did not have any tumor growth in the experimental period.

### Bone marrow-derived MSCs promote tumor cell proliferation and enhance tumor vascularization *in vivo*

**Figure 4:**
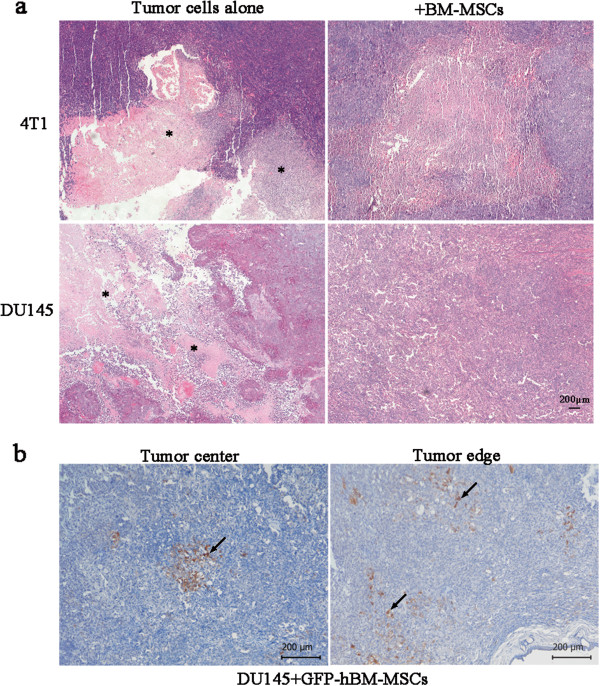
**Histological analysis of tumor sections.** (**a**) Sections from 4T1 and DU145 tumors injected with tumor cells alone or together with bone marrow-derived mesenchymal stem cells (BM-MSCs) were stained with routine H & E, and necrotic areas could be seen in the sections (*). Scale bar, 200 μm. (**b**) GFP-hBM-MSCs (arrows) could be detected by immunohistochemical analysis in paraffin sections of the DU145 + GFP-hBM-MSCs tumors collected at the end of the *in vivo* experiment with anti-GFP antibodies. Scale bar, 200 μm. hBM-MSCs, human bone marrow-derived mesenchymal stem cells.

To understand mechanisms underlying the effect of tumor growth promotion by BM-MSCs, the morphology of tumor tissue harvest at the end of *in vivo* experiments was analyzed using H & E staining. The necrosis area of 4T1 + mBM-MSC tumors and tumors from mice injected with 4T1 cells alone was compared. The staining results showed that the former had a vast area of necrosis inside the tumor tissue. However, only a limited area of necrosis could be detected in the center of 4T1 + mBM-MSC tumors (Figure 4a). Similar results were found for the DU145 tumor model (Figure 4a). To access the presence of BM-MSCs in the tumor stroma, hBM-MSCs stably expressing GFP combined with DU145 were co-injected into nude mice and the distribution of GFP-hBM-MSCs was detected by immunochemistry using anti-GFP antibody. The hBM-MSCs were randomly distributed inside the tumor but the number was relatively low (Figure 4b).

**Figure 5:**
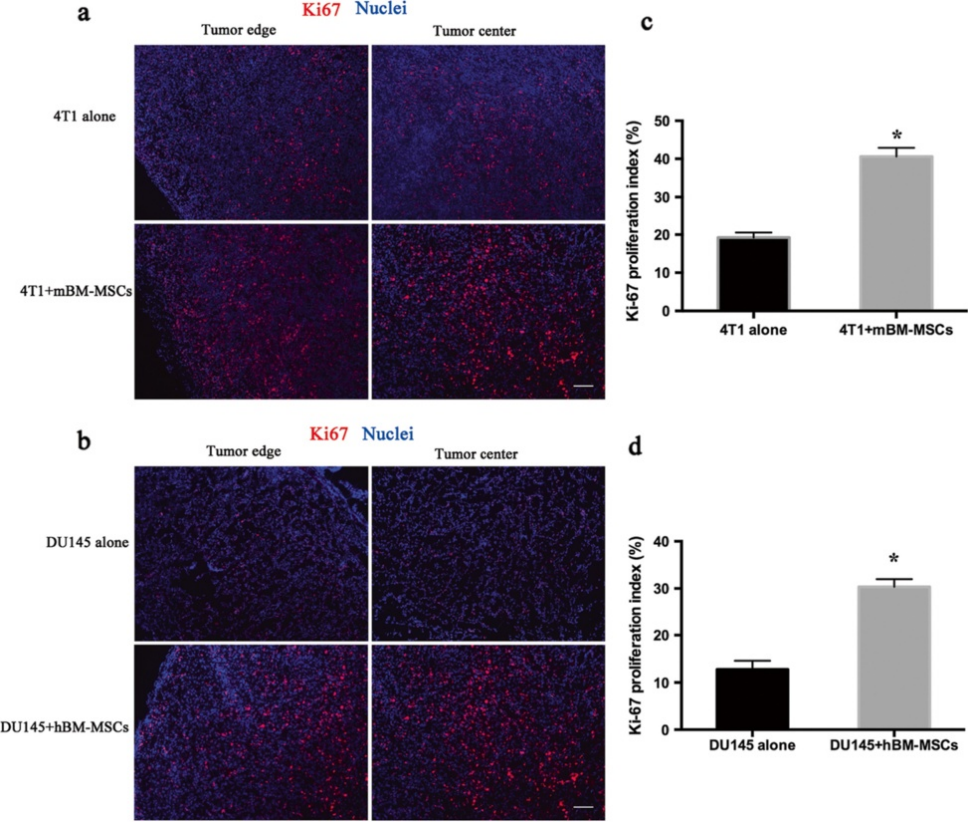
**Bone marrow-derived mesenchymal stem cells promote tumor cell proliferation *in vivo.*** Immunofluorescence analysis of proliferation cells in frozen sections from (**a**) 4T1 tumor or 4T1 combined with mouse bone marrow-derived mesenchymal stem cell (mBM-MSC) tumor and (**b**) DU145 tumor or DU145 mixed with human bone marrow-derived mesenchymal stem cell (hBM-MSC) tumor by staining with anti-Ki67. DAPI (for nuclei staining) is blue; Ki67 detected in red. Scale bar, 100 μm. Quantitative analysis of proliferation cells in (**c**) 4T1 and (**d**) DU145 tumors using the ImageJ software (*n* = 6 per group; *P <0.05).

To determine the effect of BM-MSCs on tumor cell proliferation *in vivo*, immunofluorescence was performed to detect Ki-67, a nuclear antigen widely used as a proliferation marker that is expressed by dividing cells [31]. The immunofluorescence staining results showed the majority of cells in the tumor obtained from mice injected with 4T1 + mBM-MSCs were positive for Ki-67, whereas there were less proliferation cells in the 4T1 alone group (Figure 5a). The quantification of Ki-67 proliferation index (the percentage of Ki-67-positive cells to tumor cells) revealed that the average percentage of Ki-67-positive cells in 4T1 alone tumors was 19.21%, while there was a significantly higher percentage of proliferation cells in 4T1 + mBM-MSCs tumors (40.6%; *P* <0.05) (Figure 5c). For DU145 cells we obtained consistent results. The immunofluorescence result showed that there were limited proliferation cells in the DU145 alone tumors and the number of Ki-67-positive cells was greater in DU145 + hBM-MSC tumors (Figure 5b). Average percentages of Ki-67-positive cells were 12.7% and 30.3% for DU145 alone tumors and DU145 + hBM-MSCs tumors respectively, and the difference was significant (*P* <0.05) (Figure 5d).

**Figure 6:**
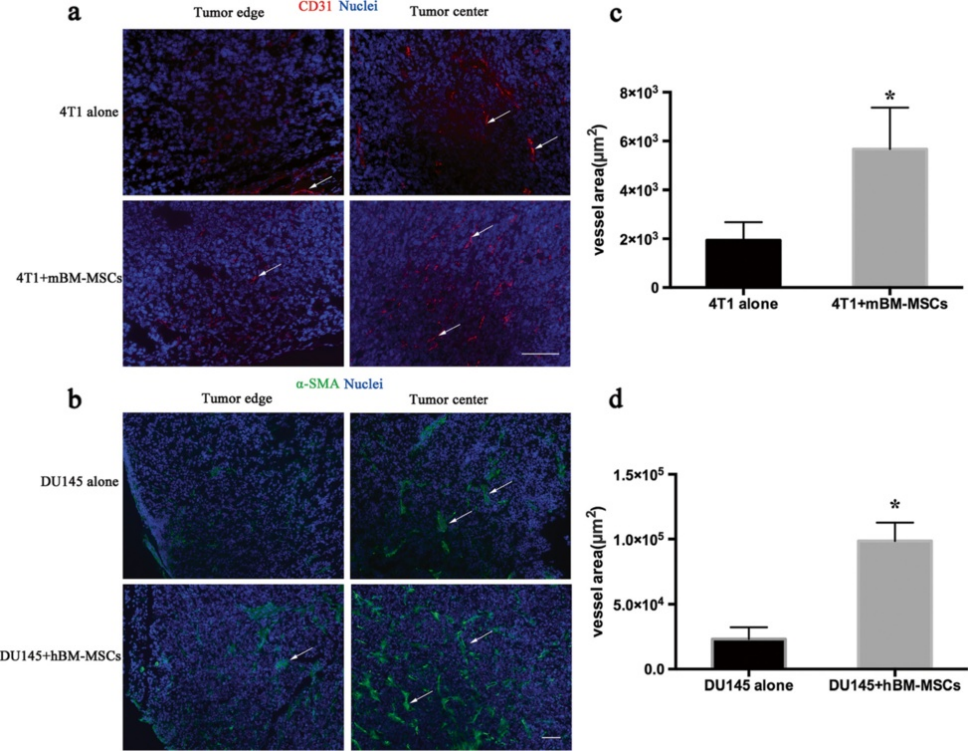
**Bone marrow-derived mesenchymal stem cells promote tumor angiogenesis *in vivo.*** Immunofluorescence analysis of blood vessel density in frozen sections from (**a**) 4T1 tumor or 4T1 combined with mouse bone marrow-derived mesenchymal stem cell (mBM-MSC) tumor by staining with anti-CD31 and (**b**) DU145 tumor or DU145 mixed with human bone marrow-derived mesenchymal stem cell (hBM-MSC) tumor by staining with anti-alpha smooth muscle actin (anti-α-SMA). DAPI (for nuclei staining) is blue; Arrows, blood vessels, Scale bar, 100 μm. Quantitative analysis of blood vessel density of (**c**) 4T1and (**d**) DU145 tumors using the ImageJ software (*n* = 6 per group; *P <0.05).

To detect the blood vessel in the tumor environment, the antibody of CD31 (platelet endothelial cell adhesion molecule-1) was used to stain the frozen tumor section by immunofluorescence to visualize the blood vessels. As compared with the 4T1 alone tumor group, the density of blood vessel in 4T1 + mBM-MSC tumors was much higher both at the edge and center of the tumor at day 20 (Figure 6a). The blood vessel density was also quantified by analyzing the percentage of CD31-positive areas. According to the quantification results, the percentage of vessel area was significantly higher in tumors from mice injected with 4T1 + mBM-MSCs than tumors injected with 4T1 alone (*P* <0.05) (Figure 6c).

For DU145 xenograft tumors, the immunofluorescence staining results revealed that the α-SMA-positive area was abundant in the center of DU145 + hBM-MSC tumors and there were also some α-SMA-positive cells present in the peripheral areas. On the contrary, the α-SMA-positive cells were much fewer both in the center and peripheral areas of the tumor in the DU145 alone group (Figure 6b). Quantification analysis showed that there was a significant increase in vessel area of tumors from mice injected with DU134 cells mixed with hBM-MSCs in comparison with tumors from mice injected with DU145 cells alone (*P* <0.05) (Figure 6d).

### Conditioned medium from human bone marrow-derived MSCs co-cultured with DU145 cells increases capillary tube formation

**Figure 7:**
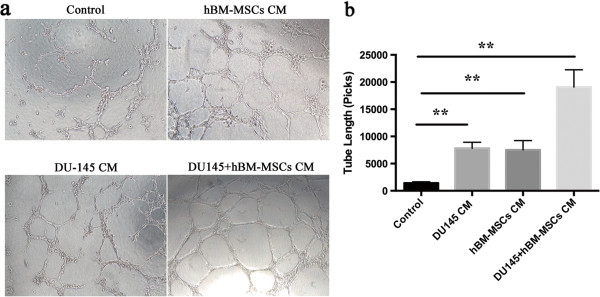
**Co-culture of human bone marrow-derived mesenchymal stem cells with DU145 cells increases tube formation.** (**a**) Representative photographs of human umbilical vein endothelial cells seeded on Matrigel in culture medium (control) and in the presence of conditioned medium from DU145 cells, human bone marrow-derived mesenchymal stem cells (hBM-MSCs) or DU145 + hBM-MSCs. Scale bar, 100 μm. (**b**) Tube length was quantitated using Image-Pro Plus software (*n* = 6 per group; **P <0.01).

To confirm our findings *in vivo* that BM-MSCs promote angiogenesis, further studies using a tube formation assay *in vitro* were performed. Conditioned medium from hBM-MSCs alone, DU145 cells alone, and co-cultures of hBM-MSCs with DU145 cells were applied in this assay. Human umbilical vein endothelial cells were incubated in normal culture medium α-MEM and were used as a control. Figure 7 shows the capillary tube formation after 8 hours in culture. By measuring the length of the capillary-like structure, the length of the endothelial cell networks was significantly increased when incubated in conditioned medium from hBM-MSCs (*P* < 0.01), DU145 (*P* < 0.01) and co-cultures (*P* < 0.01) compared with control respectively (Figure 7b). Furthermore, in comparison with conditioned medium from hBM-MSCs or DU145 alone, incubation in the co-culture conditioned medium induced a more branched network and contained enlarged cords so the ability of tube-like structure formation was dramatically enhanced (Figure 7a). Quantitative analysis also showed co-culture conditioned medium increased the tubular length to a greater extent compared with the hBM-MSCs alone and the DU145 alone groups (*P* < 0.01 vs. hBM-MSCs; *P* < 0.01 vs. DU145) (Figure 7b).

### Tumor cell conditioned medium enhances bone marrow-derived MSC angiogenic factor expression

**Figure 8:**
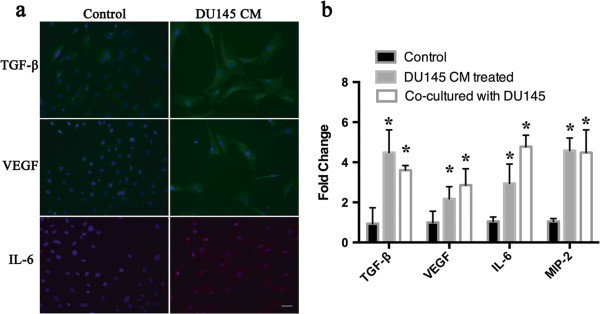
**Expression of pro-angiogenic factors by human bone marrow-derived mesenchymal stem cells.** (**a**) Immunofluorescence analysis of pro-angiogenic factor expression in human bone marrow-derived mesenchymal stem cells (hBM-MSCs) after co-culture with DU145 cells. DAPI (for nuclei staining) is blue; transforming growth factor (TGF)-β and vascular endothelial growth factor (VEGF) detected in green; IL-6 detected in red. Scale bar, 100 μm. (**b**) Real-time quantitative PCR for pro-angiogenic factor relative mRNA levels to GAPDH in hBM-MSCs, hBM-MSCs co-cultured with DU145 cells and hBM-MSCs cultured in conditioned medium of DU145 cells (*n* = 6 per group; *P <0.05). MIP-2, macrophage inflammatory protein-2.

As we observed conditioned medium from both hBM-MSCs alone and co-cultured with DU145 cells could promote the formation of capillary-like structures *in vitro*, we next analyzed the pro-angiogenic factor gene expression changes in BM-MSCs when exposed to tumor cell conditioned medium. Cell immunofluorescence staining was performed on hBM-MSCs to measure the expression of pro-angiogenic factors TGF-β, VEGF and IL-6. The results revealed there were weak TGF-β, VEGF and IL-6 staining in untreated hBM-MSCs, whereas the staining intensity increased when hBM-MSCs were exposed to DU145 conditioned medium (Figure 8a), indicating an increased production of these factors in comparison with the untreated cells. To further confirm this finding, real-time quantitative PCR was employed to detect the mRNA expression of four pro-angiogenic factors. Compared with untreated hBM-MSCs, when treated with DU145 conditioned medium or co-cultured with DU145 for 7 days there was a relative high level of all four genes (TGF-β, VEGF, IL-6 and MIP-2). The increase in mRNA level was 4.8-fold (*P* <0.05), 2.17-fold (*P* <0.05), 2.8-fold (*P* <0.05) and 4.36-fold (*P* <0.05) for TGF-β, VEGF, IL-6 and MIP-2 respectively compared with the level in untreated hBM-MSCs (Figure 8b). IL-6 was most responsive to co-cultured condition, which had 4.54-fold increase in IL-6 mRNA (*P* <0.05) compared with the control group. The mRNA expression of TGF-β, VEGF and MIP-2 was increased 3.85-fold (*P* <0.05), 2.85-fold (*P* <0.05) and 4.26-fold (*P* <0.05) respectively in the co-cultured group compared with the control group (Figure 8b).

## Discussion

Rapid tumor growth requires the recruitment of diverse stromal cells that constitute the tumor microenvironment. Paget originally proposed the concept that the stromal microenvironment plays a critical role in regulating tumor development in his ‘seed and soil’ hypothesis [32]. Among the stromal cells, CAFs communicate with cancer cells to stimulate the tumor growth and metastatic potential, and also secrete a serious of cytokine or growth factors to enhance angiogenesis [4,9]. Current evidence suggests that at least a proportion of CAFs are bone marrow derived, especially derived from bone marrow MSCs [15].

In the present study, BM-MSCs promoted tumor cell proliferation *in vitro* and tumor growth *in vivo*. However, such promotion was not associated with the number of BM-MSCs at the tumor sites. A similar finding has been reported in which MSCs promote Daudi tumor cell proliferation and protected them against apoptosis, which are partly mediated by MSC-derived soluble factors, and the maximal protective effect of MSCs on Daudi tumor cell apoptosis could be achieved by direct cell contact [33]. In contrast, Khakoo and colleagues reported that MSCs exhibit potent antitumor effects in a model of Kaposi’s sarcoma and this effect is mediated by direct cell contact leading to the inhibition of Akt activation in KS cells [28]. The different types of tumor models or sources of MSCs for assessment may be one of the factors accounting for the variability results of pro-tumorigenic or anti-tumorigenic effects. The effect of MSCs is thus context dependent and may be mediated through changes in soluble factors produced by the MSCs communicating with tumor cells in a paracrine manner [34,35].

A number of studies have demonstrated that once MSCs are incorporated into the tumor mass, they contribute with other cells such as myofibroblasts, endothelial cells, pericytes, and inflammatory cells to create a microenvironment and influence the morphology and proliferation of cells within microenvironments [36,37]. Of particular note is that angiogenesis is critical for tumor growth so that the blood vessel in the tumor environment could provide sufficient nutrients and oxygen to the cells, which are essential for the growth and survival of tumor cells [38]. According to the study of Duffy and colleagues, MSCs played active roles in angiogenesis through regulating the formation, stabilization and maturation of newly formed vessels [39]. We therefore hypothesize that enhanced angiogenesis may account for the tumor growth-promoting effects by BM-MSCs. In our study, the presence of BM-MSCs correlated with a higher abundance of blood vessels, suggesting that BM-MSCs in the tumor microenvironment contributed to promoting angiogenesis.

In addition, pericyte marker α-SMA [40] staining results suggested there was increased vascular pericyte coverage in the presence of BM-MSCs. Previous studies indicated that MSCs can function as pericyte-like cells in experimental gliomas that integrate into the tumor neovasculature [41]. In our study, the numbers of BM-MSCs detected in the tumors could not account for the increased numbers of α-SMA-positive cells. BM-MSCs may thus play a role in recruiting endogenous pericyte progenitors that participate in the formation of functional tumor vessels [42]. Furthermore, the *in vitro* effect of BM-MSCs on angiogenesis was shown in the tube formation assay*.* Collectively, these findings suggest that BM-MSCs may have the ability to potentially active angiogenesis in the tumor microenvironment. The promotion effect of MSCs on angiogenesis was consistent with a recent study by Hung and colleagues in which MSCs or factors secreted by MSCs have been shown to decrease apoptosis and enhance angiogenesis [43]. Moreover, the interaction of MSCs with tumor cells to promote angiogenesis could also be found in human ovarian carcinoma cells and melanoma cancer cells [44,45].

However, the mechanism of how MSCs stimulate angiogenesis remains to be elucidated. According to current study, we suggest that the enhancement of angiogenesis might partly attribute to the increased expression of angiogenic factors including TGF-β, VEGF, IL-6 and MIP-2 by hBM-MSCs when interacting with tumor cells. The well-established roles of these factors in promoting tumor angiogenesis strongly support our findings [46-49]. Similar to our results, Coffelt and colleagues reported that ovarian tumor-derived leucine, leucine-37 recruits MSCs to the tumor microenvironment and stimulated MSCs secreted larger amounts of pro-angiogenic factors including IL-1 receptor antagonist, IL-6, IL-10 and VEGF to support angiogenesis [50]. Tsai and colleagues demonstrated that secretion of IL-6 by MSCs activated the signal transducer and activator of transcription-3 pathway in cancer cells and promoted tumor formation [51]. Lin and colleagues reported that MSCs expressed higher levels of VEGF via the hypoxia-inducible factor-1α pathway, thus increasing tumor angiogenesis and leading to colon cancer growth in mice [52]. These previous studies provided important clues to the molecular mechanisms underlying our findings in breast or prostate tumor models. Additionally, bedsides secretion of angiogenic factors, previous studies have demonstrated that MSCs may act as a component of tumor-associated fibrovascular networks, including the pericytic population that contributes to the microvessels involved in the neovascularization as well as the fibroblastic population that contributes to matrix remodeling and tumor growth. Nevertheless, the detailed characterization of the properties of MSCs in the tumor microenvironment merits further investigation.

## Conclusion

In this study, we have demonstrated that BM-MSCs could promote tumor cell proliferation *in vitro* and tumor growth *in vivo*. The promotion effect may partly attribute to the increased expression of pro-angiogenic factors in BM-MSCs in the tumor microenvironment and subsequent enhancement in angiogenesis and tumor growth. Better understanding of the underlying mechanisms of interaction between tumor cells and MSCs could lead to establishment of new therapeutic approaches.

## Abbreviations

α-MEM: alpha-minimum essential medium; α-SMA: Alpha smooth muscle actin; BM-MSC: Bone marrow-derived mesenchymal stem cell; BSA: Bovine serum albumin; CAF: Cancer-associated fibroblast; DMEM: Dulbecco’s modified Eagle’s medium; FBS: Fetal bovine serum; GFP: Green fluorescent protein; hBM-MSC: human bone marrow-derived mesenchymal stem cell; H & E: Hematoxylin and eosin; IL: Interleukin; mBM-MSC: mouse bone marrow-derived mesenchymal stem cell; MIP-2: Macrophage inflammatory protein-2; MSC: Mesenchymal stem cell; PBS: Phosphate-buffered saline; PCR: Polymerase chain reaction; TGF: Transforming growth factor; VEGF: Vascular endothelial growth factor.

## Competing interests

The authors declare that they have no competing interests.

## Authors’ contributions

TZ participated in the design of the research, carried out the experiments of the *in vitro* and *in vivo* study, performed data acquisition and analysis, and wrote and revised the manuscript. YWL made an application for ethical approval and was responsible for collection of human fetal tissues and made critical revision of the manuscript. YFR and XHJ contributed to analysis and interpretation of the data. TYC helped with the animal experiments. GL participated in study design and coordination, data analysis and interpretation, helped to draft and gave final approval of the manuscript and financial support. All authors read and approved the final manuscript.
